# 

**Published:** 1850-01

**Authors:** 


No. VIII. JANUARY. Vol. V.

### BUFFALO MEDICAL JOURNAL AND MONTHLY REVIEW OF MEDICAL AND SURGICAL SCIENCE.

EDITED BY AUSTIN FLINT, M. D.

BUFFALO:
PUBLISHED BY JEWETT, THOMAS & CO.
Commercial Advertiser Buildings.

1850.

